# The La-related protein 1-specific domain repurposes HEAT-like repeats to directly bind a 5′TOP sequence

**DOI:** 10.1093/nar/gkv748

**Published:** 2015-07-22

**Authors:** Roni M. Lahr, Seshat M. Mack, Annie Héroux, Sarah P. Blagden, Cécile Bousquet-Antonelli, Jean-Marc Deragon, Andrea J. Berman

**Affiliations:** 1 Department of Biological Sciences, University of Pittsburgh, Pittsburgh, PA 15260, USA; 2 Photon Sciences Directorate, Bldg 745 L107 Brookhaven National Laboratory Upton, NY 11973, USA; 3 Department of Oncology, University of Oxford, Churchill Hospital, Oxford, OX3 7LE, UK; 4 CNRS-UMR5096 LGDP, 66860 Perpignan, France; 5 Université de Perpignan-UMR5096 LGDP, 66860 Perpignan, France

## Abstract

La-related protein 1 (LARP1) regulates the stability of many mRNAs. These include 5′TOPs, mTOR-kinase responsive mRNAs with pyrimidine-rich 5′ UTRs, which encode ribosomal proteins and translation factors. We determined that the highly conserved LARP1-specific C-terminal DM15 region of human LARP1 directly binds a 5′TOP sequence. The crystal structure of this DM15 region refined to 1.86 Å resolution has three structurally related and evolutionarily conserved helix-turn-helix modules within each monomer. These motifs resemble HEAT repeats, ubiquitous helical protein-binding structures, but their sequences are inconsistent with consensus sequences of known HEAT modules, suggesting this structure has been repurposed for RNA interactions. A putative mTORC1-recognition sequence sits within a flexible loop C-terminal to these repeats. We also present modelling of pyrimidine-rich single-stranded RNA onto the highly conserved surface of the DM15 region. These studies lay the foundation necessary for proceeding toward a structural mechanism by which LARP1 links mTOR signalling to ribosome biogenesis.

## INTRODUCTION

La-related protein 1 (LARP1) is implicated in the stability, localization, and translational efficiency of mRNAs required for cell development, migration, division, and general translation (1–6). LARP1 alters the association of mRNAs with polysomes and pre-polysomes, and decreases the migratory potential of cells (3,5,7). Its localization to the leading edge of cells combined with its role in translation suggests that LARP1 may play a role in directing localized protein synthesis (3). Upregulation of LARP1 occurs in several cancers, including that of the liver (8–11), cervix (12) and breast (13,14), and is correlated with tumor progression and adverse clinical outcome (12).

LARP1 plays conserved roles in regulating the stability of mRNAs across the animal and plant kingdoms. For example, in *Caenorhabditis elegans*, LARP1 localizes to P-bodies where it is thought to regulate the levels of Ras-MAPK mRNAs during oogenesis (15). Similarly, through its interaction with poly(A) binding protein (PABP), which directly binds the 3′ poly(A) tails of mRNAs, *Drosophila* LARP1 plays roles in fertility and embryonic development (2,4). This direct PABP interaction is conserved in human cells (1,3), where LARP1 has also been observed to localize to stress granules (16). In *Arabidopsis*, LARP1a participates in destabilizing mRNAs as part of the XRN4 exoribonuclease-associated heat stress response (17).

Several lines of evidence indicate that LARP1 is an RNA binding protein. While data suggests LARP1 can associate with poly-adenylate, uridylate, and guanylate sequences (1,15), it has been shown to preferentially associate with the 5′ ends of mRNAs containing terminal oligopyrimidine tracts (5′TOPs) (1,5,16). Immediately after the 5′ cap, the 5′TOP motif has 4–14 pyrimidine residues followed by a GC-rich region (18). mRNAs containing these sequences are known as TOP mRNAs and encode most translation factors and ribosomal proteins (18), which are essential for supporting cell growth and proliferation.

Accordingly, the oncogenic growth-response mTOR kinase regulates the expression of TOP mRNAs. mTOR complex 1 (mTORC1) transduces growth signals to activate the translation of ribosomal and ribosome-associated mRNAs, thereby stimulating ribosome biogenesis and cell proliferation (7). For this reason, and because of its involvement in signalling cascades that are often disrupted in cancer, mTOR and components of its pathways are attractive anti-cancer drug targets. Although mTOR inhibitors are effective in the treatment of some rare tumours, for the majority of cancers, their inability to potently target ribosome biogenesis, inhibit proliferation and cause apoptosis has limited their efficacy. Additionally, inhibition of mTORC1 causes feedback activation of PI3K/AKT, Ras/MAPK and mTORC2 signalling cascades. This results in a paradoxical increase in protein biosynthesis, cell cycling and the emergence of resistance (19–22).

Although mTORC1 signalling regulates TOP mRNA translation, neither mTOR itself, nor any of the subunits within its active complexes (mTORC1 and mTORC2), has been shown to directly interact with 5′TOPs (18). Recent data have identified LARP1 as an mTORC1 substrate that binds 5′TOPs to enhance their translation (5,18) and as a target of mTOR kinase through mutual association with the Raptor subunit of mTORC1 (5,16). This positions LARP1 as the integrator in the mTOR-TOP signalling axis and directly links it to ribosome biogenesis (19–22). Further, its downstream location within the mTORC1 cascade and its direct control over proliferation via TOP regulation (5) makes LARP1 a potential anti-cancer target.

La and its related proteins, so named because they carry an eponymous ‘La motif’ that adopts a winged helix-turn-helix fold (23–26), exist in all eukaryotes and can be divided into subfamilies based on the conservation of sequence beyond the La motif. Most of these subfamilies retain at least one RNA recognition motif (RRM) C-terminal to the La-motif (27). LARP1, the largest member of the LARP family, contains an RRM with a predicted βαββαβ fold, having characteristic secondary structural element lengths conserved across all LARP1 proteins (27). LARP1 also includes a highly conserved LARP1-specific region in its C-terminus that contains at least one DM15 box (also referred to as a unit, motif, module or repeat) (15,27,28). These units are well conserved among species, with at least 50% similarity in amino acid sequence across more than 90% of the identified LARP1 proteins (27). Until now, the DM15 region in human LARP1 had been predicted to be comprised of two such units, boxes, or modules (A and B) that are present in LARP1 proteins throughout evolution and have most likely arisen through duplication (27). Other organisms, including plants, contain three DM15 modules, denoted A, B and C.

Here, we present the first evidence that the DM15 region of human LARP1 mediates its RNA binding activity. We also present the crystal structure of the DM15 region from human LARP1, the first structure from LARP1, which provides evidence for its RNA-binding capabilities. Our crystal structure reveals three structural repeats that correspond to three DM15 modules, which we have found to exist in nearly all LARP1 protein sequences. Importantly, because the expression of LARP1 is upregulated in several cancers and the conserved C-terminal LARP1-region is predicted to be exclusive to LARP1, our crystal structure reveals a unique target for future drug design studies.

## MATERIALS AND METHODS

### Protein cloning, expression and purification

The DM15 region (DM15) of the LARP1 coding sequence (amino acids 796–946 from isoform LARP1a) used in x-ray crystallography and biochemical studies was cloned by PCR from full-length LARP1 coding sequence (accession number BC001460.2) into a modified pET28 vector. The resulting construct expressed DM15 with an N-terminal His_6_-MBP tag followed by a Tobacco Etch Virus protease cleavage site and glycine-linker. Plasmids expressing point mutants of DM15 were generated using site-directed mutagenesis.

Expression plasmids were transformed into BL21(DE3) *Escherichia coli* cells and grown overnight on LB agar plates supplemented with 30 μg/ml kanamycin. The His_6_-MBP-DM15 fusion protein was expressed by scraping a ∼75% confluent plate of transformed *E. coli* into 750 ml autoinduction media (29), culturing for 3 h at 37°C, and then moving cultures to 18°C for 18 h. Cells were collected, flash frozen in liquid nitrogen, and stored at −80°C.

Cells were resuspended in lysis buffer (50 mM Tris–HCl, pH 7.5, 400 mM NaCl, 10 mM imidazole, 10% v/v glycerol) with Complete EDTA-free protease inhibitor tablets (Roche) and 4 mg ml^−1^ lysozyme. Cells were lysed by a combination of freeze-thaw and homogenization, and the resulting lysate was clarified by centrifugation. His_6_-MBP-DM15 was purified in batch by nickel agarose affinity chromatography (ThermoScientific) and eluted with 50 mM Tris–HCl, pH 7.5, 400 mM NaCl, 250 mM imidazole, 10% glycerol. The His_6_-MBP tag was removed by 2 mg TEV protease digestion overnight at 4°C during dialysis into 50 mM Tris–HCl, pH 8, 150 mM NaCl, 0.5 mM EDTA, 10% glycerol. Nucleic acid and protein contaminants were removed by tandem HiTrap Q and HiTrap SP ion exchange (GE Healthcare Lifesciences); DM15 free of nucleic acid contamination was eluted off the HiTrap SP column with a NaCl gradient (150 mM–1 M) over 10 cv. The fractions containing DM15 were pooled and brought to 1 M ammonium sulfate, 50 mM Tris–HCl, pH 7 and loaded onto a 5 ml butyl HP column (GE Healthcare Lifesciences). The butyl HP column was eluted in 50 mM Tris–HCl, pH 7, 2 mM DTT. The fractions containing DM15 were concentrated and buffer exchanged using a 10K MWCO concentrator in 50 mM HEPES, pH 7, 2 mM DTT to a final concentration of 20 mg ml^−1^ for crystallography or in 50 mM Tris–HCl, pH 7.5, 250 mM NaCl, 25% glycerol, 2 mM DTT to a final concentration of 2 mg ml^−1^ for biochemistry. Point mutants of DM15 were purified and stored in the same manner as WT.

Selenomethionine-derivatized DM15 (Se-met) autoinduction (29) and protein purification were performed as described above, with the addition of 2 mM DTT throughout the prep after nickel affinity chromatography. Se-met protein was concentrated to 18 mg ml^−1^.

### Crystallization and structure solution

DM15 crystals were grown by hanging drop vapor diffusion at room temperature overnight using a 1:1 protein to mother liquor ratio against a reservoir solution of 0.15 M sodium thiocyanate, 17% PEG 3350 (optimized from JCSG+ [Qiagen] condition 14). Native crystals were cryoprotected by equilibration to 0.15 M sodium thiocyanate, 25% PEG 3350, 10% glycerol.

Se-met crystals were grown by hanging drop vapor diffusion at room temperature for one week using a 1:1 protein to mother liquor ratio against a reservoir solution of 0.2 M ammonium nitrate, 20% PEG 3350 (optimized from JCSG+ [Qiagen] condition 27). Se-met crystals were cryoprotected by equilibration to 0.2 M ammonium nitrate, 25% PEG 3350.

X-ray crystallographic data was collected at the National Synchrotron Light Source (Brookhaven National Lab). Single-anomalous dispersion of Se-met crystals was used to phase the structure in Phenix (30). Non-crystallographic symmetry averaging combined with solvent flattening was then used to generate an interpretable map (30). Models were built with Coot (31) and refined with Phenix (30). Finally, the resolution of the derivative data was extended by using the experimentally derived structure as a search model for molecular replacement (30) into a native dataset that was truncated at 1.86 Å resolution. Simulated annealing composite omit maps were used to confirm amino acid register (Phenix (30), CCP4 (32)). Iterative building and refinement were performed in Coot (31) and Phenix (30), respectively. Figures were generated with the PyMOL Molecular Graphics System (Schrödinger, LLC.).

### RNA sequences

RNA oligonucleotides used in the biochemical assays contained the following 5′-3′ sequences: A_16_ (AAAAAAAAAAAAAAAA) (Sigma), U_16_ (UUUUUUUUUUUUUUUU) (Sigma), RPS6–20mer (CCUCUUUUCCGUGGCGCCUC) (Sigma), RPS6–42mer (CCUCUUUUCCGUGGCGCCUCGGAGGCGUUCAGCUGCUUCAAG) (T7 *in vitro* transcribed), RPS6 ΔTOP (GUGGCGCCUCGGAGGCGUUCAGCUGCUUCAAG) (T7 *in vitro* transcribed), PABPC1 (CCCCUUCUCCCCGGCGGUUA) (Sigma), RPL13A (CACUUCUGCCGCCCCU) (IDT).

### Electrophoretic mobility shift assays

RNA oligos were 5′-end labelled with [γ-^32^P]-ATP (Perkin Elmer) using T4-polynucleotide kinase (New England Biolabs) and gel purified. 5X protein stocks were prepared in protein dilution buffer (50 mM Tris–HCl, pH 7.5, 250 mM NaCl, 25% glycerol, 2 mM DTT). Binding reactions contained final concentrations or amounts of 20 mM Tris–HCl, pH 8, 150 mM NaCl, 10% glycerol, 1 mM DTT, 0.5 μg tRNA (Life Technologies), 1 μg BSA and <2 nM RNA. Wild-type DM15, R840E and Y883A proteins were titrated at 0, 0.01, 0.03, 0.1, 0.3, 1, 3, 10, 30 μM; GQ908–909AA protein was titrated at 0, 0.5, 1, 5, 10, 20, 50, 100, 150 μM. Reactions were incubated on ice for 30 min and loaded on 7–8% polyacrylamide (29:1) native 0.5× TBE gels at 4°C. Gels were run at 120 V for 40 min at 4°C, dried, and exposed overnight. Exposed phosphor screens (GE Healthcare Lifesciences) were imaged on a Typhoon FLA plate reader (GE Healthcare Lifesciences) and quantitated using Imagequant TL (GE Healthcare Lifesciences). Fraction shifted was calculated by taking the ratio of the sum of the background-corrected volume intensities for all bands above the unshifted probe over total counts per lane.

### Sequence alignments

Sequences were aligned using the multiple sequence comparison by log-expectation (MUSCLE v3.7) software (33) and represented using JalView (34).

### Cross-linking

DM15 was incubated with trace amounts of radiolabelled oligonucleotide in 150 mM NaCl, Tris–HCl, pH 8, 1 mg ml^−1^ BSA and exposed to 254 nm UV light for 30 min or over a time course of 0–90 min. Each crosslinking reaction was analysed by SDS-PAGE on a 15% polyacrylamide gel (37.5:1) and visualized by phosphor screen.

### Size exclusion chromatography

All samples were run and absorbance data (280 and 254 nm) recorded on an Akta Pure chromatography system (GE Healthcare Lifesciences). Samples (90 μg DM15; 23 μg DM15 + 7.4 μg RPS6 oligonucleotide (1:1 molar ratio) pre-incubated on ice for 20 min; 7.4 μg RPS6 oligonucleotide; or molecular weight standards (BioRad)) were analysed with a HiLoad 16/600 Superdex 75 pg column (GE Healthcare Lifesciences) in 50 mM Tris–HCl, pH 8, 200 mM NaCl, 1 mM DTT, 2% glycerol.

### Nuclease probing

RNA oligos were 5′ end labelled with [γ-^32^P]ATP (Perkin Elmer) using T4-polynucleotide kinase (NEB) and gel purified. 5X protein stocks were prepared in protein dilution buffer (50 mM Tris–HCl, pH 7.5, 250 mM NaCl, 25% glycerol, 2 mM DTT). All reactions contained 0.5 μg tRNA (Life Technologies), 1 μg BSA, and 10 000 counts/μl radiolabelled RNA. Binding reactions were incubated at room temperature for 30 min prior to the addition of RNase. Digestion reactions were conducted for 10 min at room temperature and were stopped with formamide loading dye and loaded directly on 12% polyacrylamide (29:1) 1× TBE 7M urea gels, which were run at 90 W for 45 min. Gels were dried, exposed overnight and phosphor screens (GE Healthcare Lifesciences) were imaged with a Typhoon (GE Healthcare Lifesciences).

RNaseT1: 5′-end labelled RNA was incubated with DM15 (0–20 μM) for 30 min at room temperature prior to the addition of 0.1 unit of RNaseT1 (ThermoScientific).

RNase A: 5′-end labelled RNA was incubated with 0 or 10 μM DM15 for 30 min at room temperature. Serial dilutions of RNase A (ThermoScientific) were tested for each reaction: 0, 0.001 ng, 0.005 ng, 0.01 ng, 0.05 ng, 0.1 ng.

The hydroxyl RNA ladder was generated by incubating 10 000 counts of 5′ end-labelled RNA in 50 mM sodium carbonate, pH 11.7, 1 mM EDTA. Reactions were heated at 95°C for 4 min and incubated on ice for 5 min. The RNase T1 ladder was generated by digesting 10 000 counts of 5′ end-labelled RNA in 25 mM sodium citrate, pH 5.5, 7M urea with 2 units of RNase T1 at 50°C for 20 min.

## RESULTS

### The human DM15 motifs interact directly with RNA

**Figure 1.**
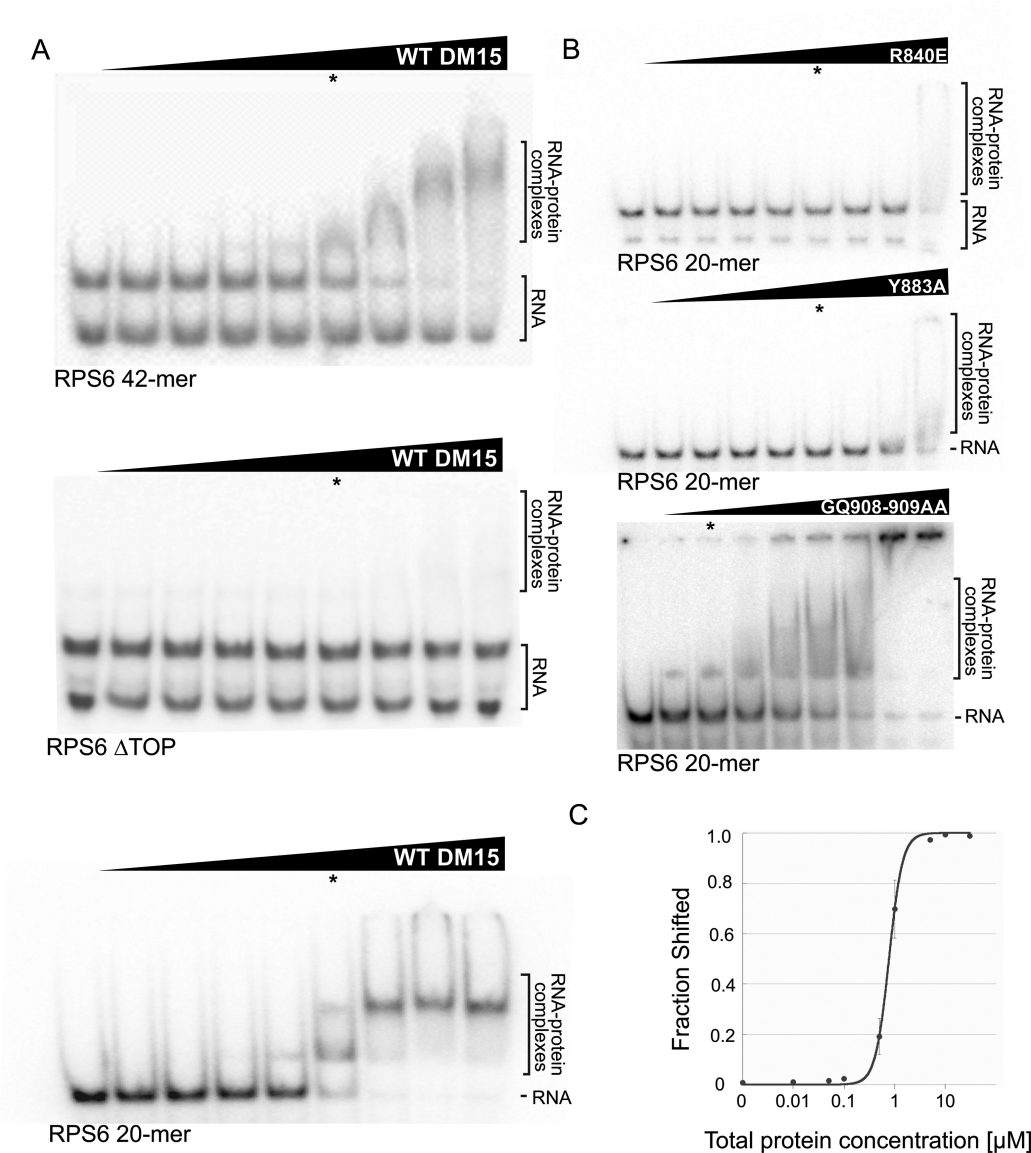
DM15 binds the 5′TOP sequence of RPS6. (**A**) Representative EMSAs of WT DM15 with oligonucleotides containing the first 42 nucleotides of the RPS6 5′TOP (RPS6-42mer; top), the RPS6 TOP lacking the 5′ polypyrimidine tract (RPS6 ΔTOP; middle), and the first 20 nucleotides of the RPS6 5′TOP (RPS6-20mer; bottom). (**B**) EMSAs of point mutants of DM15 at the putative DM15 RNA-binding and non-crystallographic dimerization surfaces. RNA binding of these point mutants was tested with the RPS6-20mer oligonucleotide. (**C**) Quantification of three independent EMSAs of wild-type DM15 bound to radiolabelled RPS6-20mer RNA oligonucleotide; error bars show standard deviation. In (A) and (B), asterisks denote 1 μM total protein for reference.

The DM15 region (DM15) of LARP1 has highly conserved motifs of unknown function. While several publications have reported an RNA binding activity of the C-terminal conserved region of LARP1 (1,15), the methods utilized were complicated by the presence of other cellular components. To overcome this, we conducted direct *in vitro* RNA binding assays of the human DM15 using amino acids 796–946 from the LARP1a isoform (corresponding to amino acids 873–1023 in the longer isoform 2 (13) of LARP1) (Figure 1A and B). Since LARP1 has been implicated in binding both the 5′ and 3′ ends of mRNA targets, we tested an oligonucleotide representing the 3′ poly(A) tail and RNA oligonucleotides from the 5′ untranslated region of the mRNAs encoding RPS6 (1), RPL13A and PABPC1 (5). These 5′TOPs had previously been identified as LARP1 interactors (1,5,16). We also tested the cognate poly-U RNA binding sequence of genuine La protein, the first identified and best studied La-motif containing protein (35).

Electrophoretic mobility shift assays conducted with an oligonucleotide representing the 5′TOP sequence from RPS6 (RPS6 42-mer) demonstrate that DM15 directly binds this sequence in two concentration-dependent complexes (see below). DM15 did not interact with a related olignonucleotide lacking the polypyrimidine tract (RPS6 ΔTOP), suggesting DM15 specifically recognizes at least the 5′ pyrimidine-rich region of this sequence (Figure 1A). These data also suggest that the DM15 region largely supports the LARP1 interaction with the RPS6 5′TOP reported by Fonseca *et al*. (16).

DM15 did not bind the oligonucleotides representing the first sixteen or twenty nucleotides of the 5′TOPs from RPL13A or PABPC1 (data not shown), respectively. The RPL13A and PABPC1 sequences have a shorter and longer polypyrimidine stretch, respectively, compared to that in the RPS6 (5) and RPL32A (16) sequences. These observations might hint at an optimal polypyrimidine length for DM15 recognition.

RNase probing assays reveal that DM15 protects nucleotides CUUUUC and GGCG of the RPS6 5′TOP sequence, expanding its recognition motif beyond the polypyrimidines (Supplementary Figure S1). Consistent with this observation, DM15 cannot shift shorter oligonucleotides representing only the first 8 or 11 nucleotides of the RPS6 5′TOP sequence (data not shown), but can shift a 20-mer (Figure 1A), suggesting a requirement for the 3′ G-rich end of this motif. Quantification of the interaction of the DM15 construct with this 20-mer oligonucleotide reveals an affinity of 776 ± 26 nM (∼500 nM when corrected for active protein concentration) in the presence of non-specific competitor molecules (Figure 1C). No specific binding was observed for either the poly(U) (U_16_) or the poly(A) (A_16_) oligonucleotide (data not shown). UV and glutaraldehyde cross-linking experiments and analytical gel filtration suggest that one or two molecules of DM15 can interact with one RNA molecule comprised of 16–20 nucleotides (Supplementary Figure S2 and data not shown).

### The DM15 region contains three helix-turn-helix HEAT-like repeats

To understand the mechanism of its RNA binding activity, we determined the crystal structure of DM15 from human LARP1 to 1.86 Å resolution (Table 1), using the same construct we used in the binding studies. The crystal structure contains four copies of DM15 in the asymmetric unit in two pseudo-dimers; ∼98% of the amino acids are ordered and visible in the experimental map. Superposition of all four copies based on Cα positions yields a range of root-mean-square deviation (RMSD) among six pair-wise permutations of 0.29–0.95 Å (Supplementary Figure S3), with the majority of the differences appearing within the C-terminus of the construct.

**Table 1.** Data collection, phasing and refinement statistics

	Se-Met	Native
**Data collection**		
Space group	*P*2_1_	*P*2_1_
Unit cell dimensions		
*a*, *b*, *c* (Å)	76.7, 61.5, 83.2	77.9, 61.8, 85.3
*β* (°)	115.5	116.2
Resolution (Å)	50–1.8	50–1.62
*R*_merge_ (%)	9.6 (9.1)	15.3 (10.8)
*I*/σ(*I*)	34.2 (2.25)	34.6 (1.06)
Completeness (%)	99.9 (99.9)	94.7 (63.7)
Redundancy	6.4 (3.0)	5.4 (2.4)
Wavelength (Å)	0.979	1.10
Unique reflections	35 696	87 976

**Phasing**		
Method	SAD^a^	MR^b^
Resolution range	42.6–2.2	38.3–1.86
No. sites/copies^c^	8	4
FoM^d^	0.484	N/A

**Refinement**		
Resolution (Å)	42.6–2.20	38.3–1.86
*R*_work_/*R*_free_	17.4/22.2	17.9/21.5
RMSD bond angle (°)	0.991	0.921
RMSD bond length (Å)	0.008	0.007
Average *B*-factor (Å^2^)	31.5	25.7
PDB ID	5C0V	4ZC4

^a^SAD: single wavelength anomalous dispersion.

^b^MR: molecular replacement.

^c^number of selenium sites found (SAD) OR number of search models placed (MR).

^d^FoM, figure of merit (SAD).

**Figure 2.**
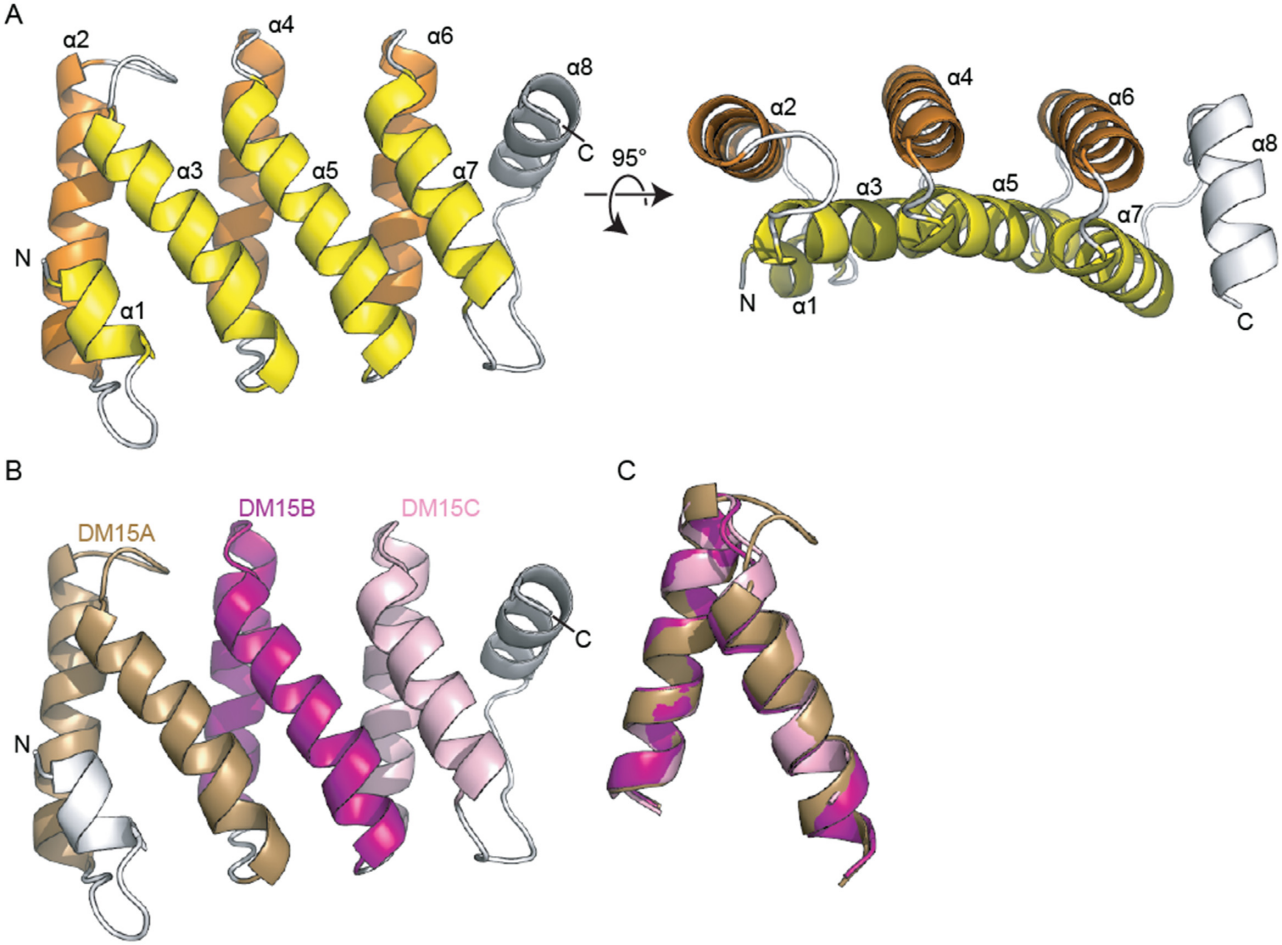
The human DM15 region contains three helix-turn-helix repeats. (**A**) Ribbon diagram of the structure of LARP1 amino acids 796–946. The eight alpha helices observed are labelled numerically from N- to C-terminus. The two layers of helices are coloured yellow and orange; the loops and the helix (α8) that does not lie parallel to other helices are shown in white. The left panel is related to the right panel by a rotation about the x-axis of ∼95°. (**B**) The DM15 region contains three helix-turn-helix repeats. DM15A is shown in brown, DM15B in purple, DM15C in pink. (**C**) Alignment of three structural repeats, coloured as in (B).

DM15 of human LARP1 folds into two layers of alpha helices, with each layer containing three or four parallel alpha helices whose axis is offset from the helical axis of the other layer by ∼40° (Figure 2) (36). The helices partition into layers in an alternating fashion, with the even helices forming one layer, and the odd helices forming the second layer; the C-terminal helix of this construct, α8, lays approximately orthogonally to both layers. An edge-on view of the molecule reveals that the helices pack into a curved structure, creating concave and convex surfaces (Figure 2A, right). The monomer appears to have a pseudo-two-fold symmetrical organization about its center, although the capping helices do not conform to this pseudo-symmetry. One of the four copies of the molecule in the asymmetric unit has a four-residue helical turn between α7 and α8 (Supplementary Figure S3).

A search of the structural database with the DALI server (37) reveals that the helix-turn-helix repeats contained within DM15 are structurally similar to tetratricopeptide repeats (TPRs). However, the angles between sequential helices differ in DM15 as compared to the angles observed in ideal TPRs (38) and the sequence of each repeat is inconsistent with the consensus sequence of TPRs (39). Likewise, the sequences of these repeats do not align with the consensus sequences of other helix-turn-helix repeats, like HEAT repeats (40) or pentatricopeptide repeats (41). Additionally, the lengths of the repeats in DM15 are inconsistent with the lengths of repeats in these modules. However, at least two of the three structural repeats in DM15 appear to contain a kinked-alpha helix, consistent with HEAT repeats.

### Sequence conservation of the third DM15 box

The human DM15 region of LARP1 was predicted to contain two evolutionarily conserved amino acid sequences, DM15A and DM15B (27). Sequence assignment of the alpha helices indicates that the conserved DM15A element occupies α2 and α3 and the DM15B element lies within α4 and α5 (Figure 2B). Helices 6 and 7 form a structural repeat that packs against the DM15A and B repeats and contains elements of both the DM15A and DM15B consensus sequences (Figure 2B,C). Therefore, human LARP1 contains three DM15 boxes.

**Figure 3.**
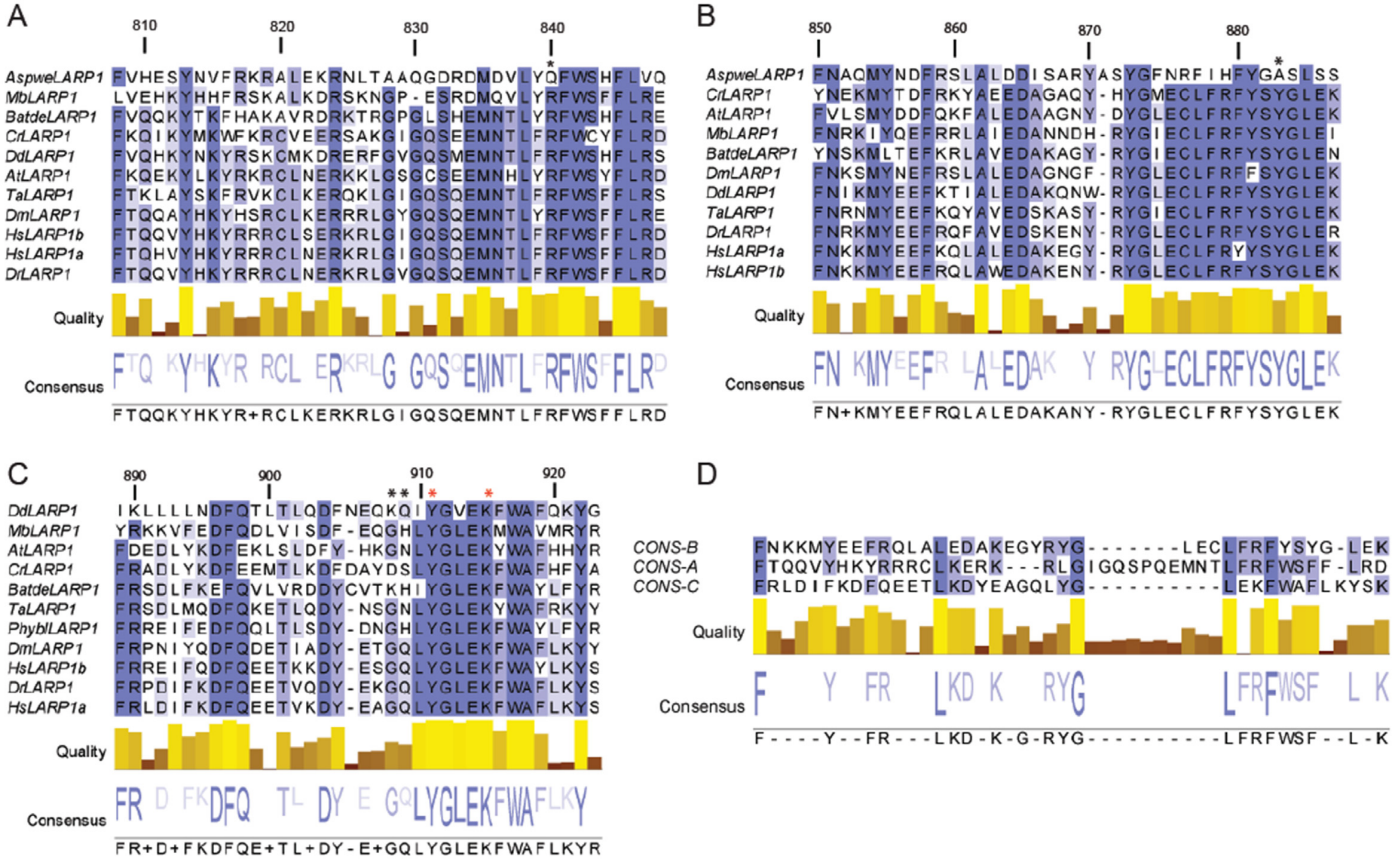
Alignment of the LARP1 DM15 region from plant, protista, fungi and animal species. Alignment of the (**A**) DM15A, (**B**) DM15B and (**C**) DM15C repeats from select plant, protist, fungi and animal species. (**D**) Alignment of the DM15 A, B and C consensus sequences. Black asterisks, amino acid targeted by mutagenesis (Figure 4). Red asterisks, amino acids that mediate dimer interactions through the co-crystallized sulphate ion (Figure 5). The numbers above each alignment indicate the position in the human LARP1a sequence. The intensity of the sequence colouring is proportional to the level of conservation. The species code is the following: **Animal:** Dm: *Drosophila melanogaster*, Dr: *Danio rerio*, Hs: *Homo sapiens*, Ta: *Trichoplax adhaerens*. **Fungi:** *Aspwe: Aspergillus wentii*, *Batde: Batrachochytrium dendrobatidis, Phybl: Phycomyces blakesleeanus*.**Plant and algae:** At: *Arabidopsis thaliana*, Cr: *Chlamydomonas reinhardii*. **Protista:** Mb: *Monosiga brevicollis*, Dd: *Dictyostelium discoideum*. A more complete alignment of the LARP1 DM15 region is shown in Supplementary Figure S4.

Having observed these three structural DM15 repeats in human LARP1, we examined the conservation of these repeats across evolution (Figure 3, Supplementary Figure S4). We aligned the DM15 region of LARP1 proteins from 71 representative plant, protist, fungi and animal species (Supplementary Figure S4). We observed that the third DM15 repeat of the human protein is highly homologous to the DM15C box, previously proposed to be specific to plants (27). In fact, in all eukaryotes, except for Ascomycota fungi, LARP1 possesses three related, well-conserved, DM15 boxes (A–C). Sequence alignment of each box independently (Figure 3A–C), and of the three consensus sequences (Figure 3D), strongly suggests that these elements evolved in a concerted manner from a single common ancestral motif. Sequence conservation in the DM15 region is centered on the three DM15 repeats, with short N- and C-terminal extensions that encompass the first and last helices found in the human protein. Although Ascomycota fungi LARP1 do not present a conserved DM15C element and possess less well-conserved DM15A and B elements, this DM15 region is still predicted to fold in a similar fashion to the human protein (data not shown), suggesting a conserved structural organization.

### Conserved residues map to a positively charged surface

**Figure 4.**
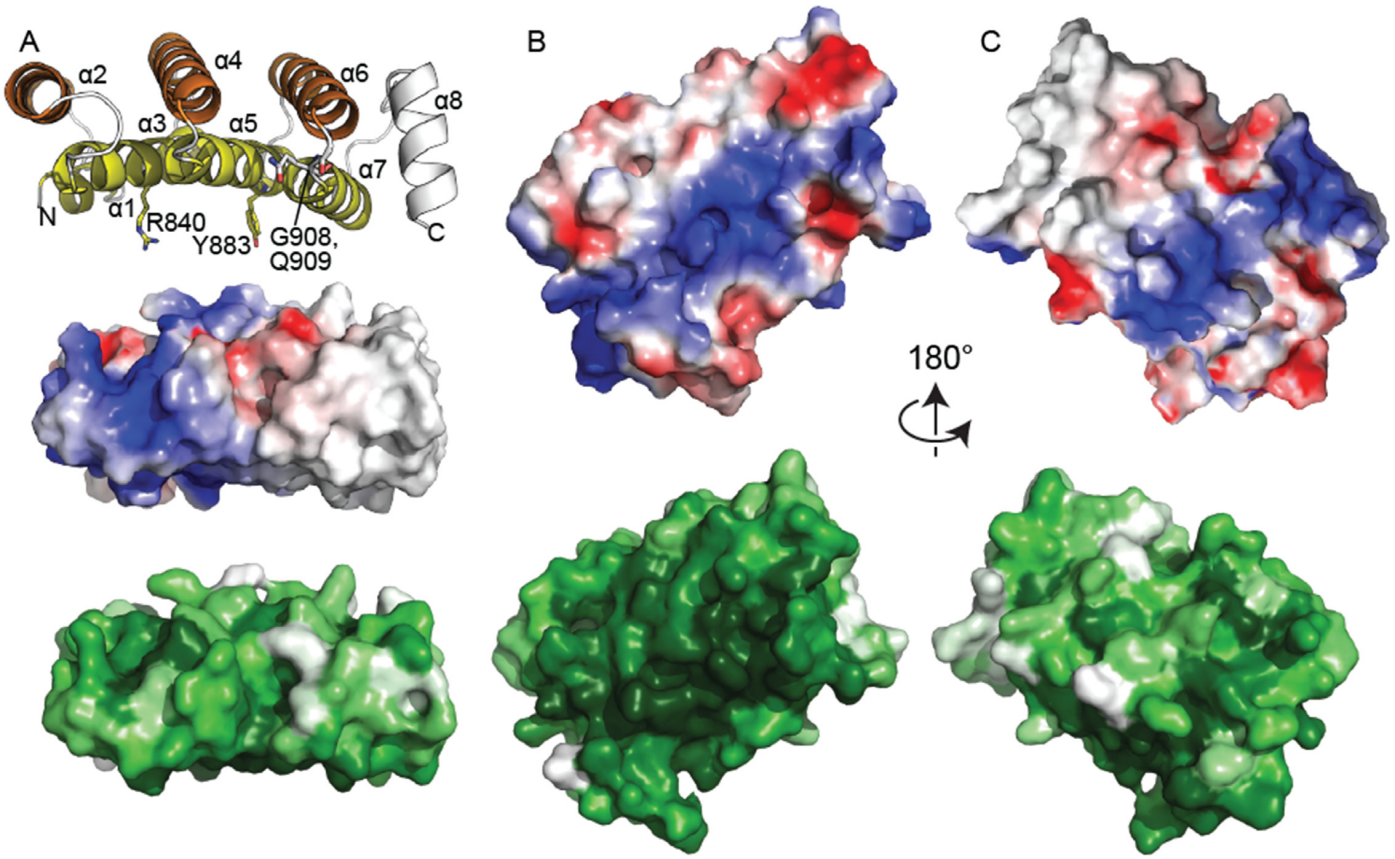
The concave surface of DM15 is conserved and positively charged. (**A**) Edge-on views of DM15 reveal that it assumes a curved structure. Top, cartoon representation and coloured as in Figure 2A. Amino acids tested for their roles in binding the RPS6 5′TOP oligonucleotide are shown as sticks. Middle, surface electrostatic representation as calculated by PyMOL (blue, electrostatically positive; red, electrostatically negative). Bottom, surface representation coloured by conservation as calculated by Consurf (42) using alignment of DM15 sequences from Supplementary Figure S4 (white, less conserved; dark green, more conserved). (**B**) View of the concave DM15 surface, coloured as in (A). (**C**) View of the convex DM15 surface, coloured as in (A). The view in panel (B) is related to that in panel (C) by a 180° rotation about the x-axis.

Electrostatic profile calculations mapped onto the surface representation of the crystal structure of DM15 reveal two positively charged patches (Figure 4), one on each side of the curved molecule. One of these patches contains several amino acids that are identically conserved in 90–100% of LARP1 proteins examined (27,42) (see Supplementary Figure S4). This patch resides on the concave side of the molecule, the macromolecular surface of solenoidal and curved structures containing helix-turn-helix repeats (39,40,43) that typically supports intermolecular interactions. Biochemical analysis of conserved amino acids in the patch on the concave side is consistent with their participation in RNA binding; mutations R840E and Y883A obliterate RNA binding (Figures 1, 3 and 4).

### The DM15 region is a monomer in solution

**Figure 5.**
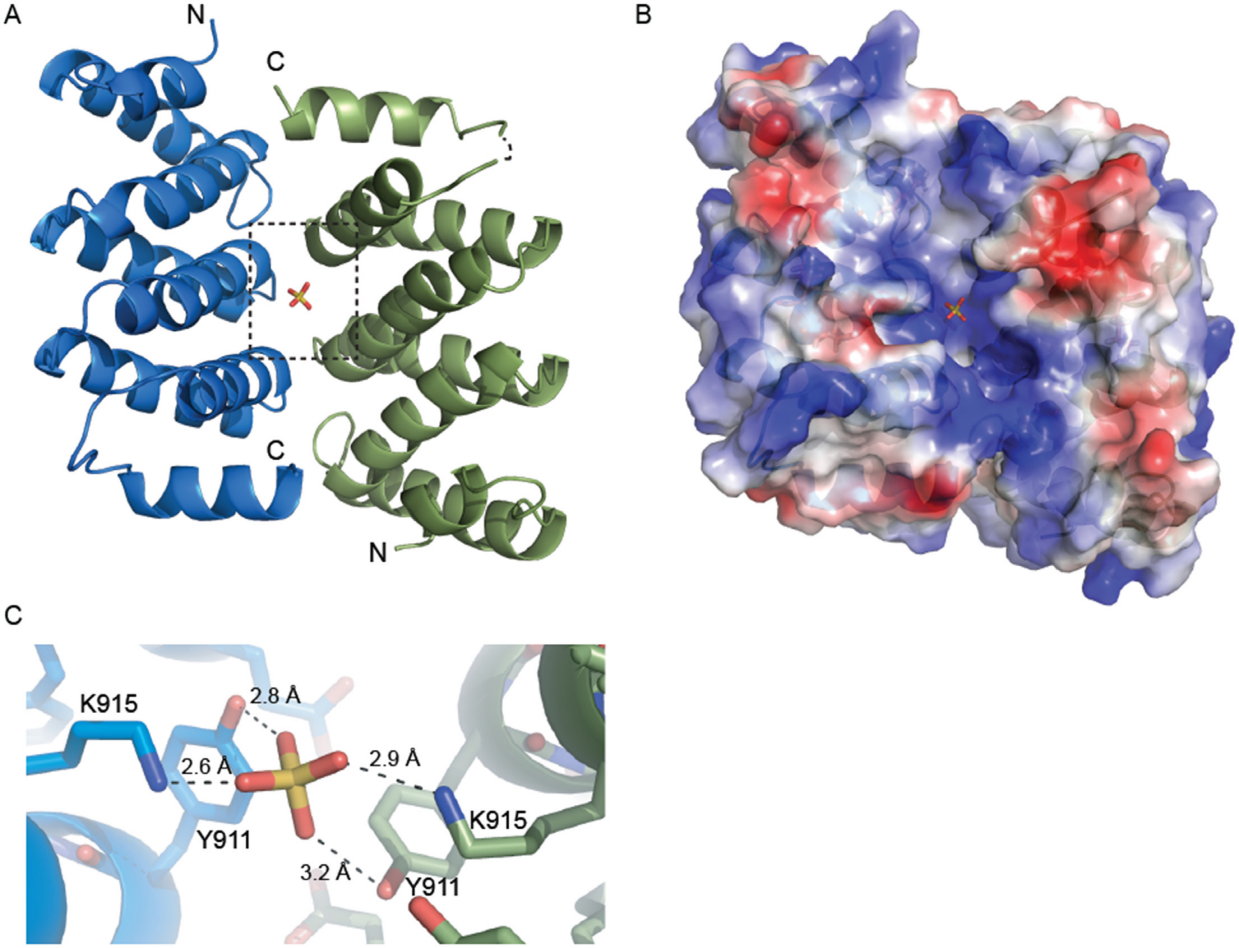
Dimerization of DM15 in the crystal. (**A**) A pseudo-twofold axis of rotation passes through the co-crystallized sulfate ion. The N- and C-termini of each monomer are labelled. (**B**) A surface representation of the same view as in (A), coloured according to electrostatic potential. A positively charged cleft is generated upon dimerization of DM15. (**C**) A zoomed view of the area in (A) bordered by the dotted box. The bound sulfate ion interacts directly with K915 and Y911 on both molecules within the non-crystallographic pseudo-dimer.

The four copies of DM15 in the asymmetric unit partition into two ‘V’-shaped dimers. A sulfate ion mediates each dimer through direct, symmetric interactions with conserved Tyr911 and Lys915 (Figures 3 and 5) of each molecule. Thus, a pseudo-twofold axis of symmetry passes through each sulfate ion approximately perpendicular to the plane of the α8 helices. The more highly conserved surface of each monomer faces the symmetry axis, forming a deep, positively charged cleft in which the co-crystallized sulfate ion binds. The two dimers superimpose with an RMSD of 0.806 Å over 282 Cαs (Supplementary Figure S3).

The two monomers of DM15 interact through more than thirty hydrophobic, ionic and hydrogen-bonding interactions (Supplementary Figure S5), several of which involve conserved amino acids (44,45) and are pseudo-symmetric. For example, the side chain of Q909, conserved in animals and a polar residue in other organisms (Figure 3, Supplementary Figures S4 and S5), interacts with the backbone chemical groups of Gly869 and Arg871 in each molecule. In addition, the side chain of Tyr872 from one copy interacts with the side chain of Leu910 in the other copy (Figure 3, Supplementary Figure S5).

We next probed the biological relevance of this dimerization interface. As mentioned above, mutation of amino acid Arg840 or Tyr883 eliminates RNA binding (Figure 1B); based on their positions relative to the dimerization interface (Supplementary Figure S5), Tyr883 most likely participates in RNA binding, whereas Arg840 might participate in both RNA binding and dimerization. A construct with alanine substitutions for two amino acids that reside at the dimerization interface but not near the predicted RNA binding site (G908 and Q909; Supplementary Figure S5F), retains RNA binding and has a two-state shift profile similar to that of wild-type DM15, presumably representing the 1:1 and 1:2 RNA:protein stoichiometries (Figure 1B); this mutant was less stable upon purification and required more protein to achieve a maximum probe shift (see methods).

We also directly assessed the oligomeric state of wild-type DM15. In the absence of RNA, DM15 elutes in a size exclusion chromatography experiment as a monomer, even in the presence of sulfate ion (data not shown). However, more than one RNA-bound complex elutes in the presence of the RPS6 5′TOP (Supplementary Figure S2). Consistent with this observation, UV and glutaraldehyde crosslinking conducted over a range of protein concentrations suggest that one or two molecules of DM15 can bind a single molecule of RNA (Supplementary Figure S2 and data not shown). Additionally, RNase footprinting of DM15 bound to the RPS6 5′TOP 20-mer oligonucleotide reveals 6 and 4 protected nucleotides, separated by three unprotected nucleotides (Supplementary Figure S1); based on the dimensions of DM15, we predict that fewer than eight nucleotides can bind its concave surface, suggesting that two molecules of DM15 might bind a single RNA substrate at higher protein concentrations. Alternatively, two populations of DM15-RNA might exist, with one site protected in one population and the other site protected in the second population.

Therefore, our data are consistent with a monomeric DM15 in the absence of RNA and the ability of up to two molecules of DM15 to bind the RNA oligonucleotides tested. However, we cannot exclude the possibility that LARP1 is a dimer in solution or in the cell. Additionally, it remains to be determined if there is a concentration dependence on the LARP1:RNA stoichiometry in the cell, since in cancer cells, in particular, there is a significant increase in LARP1 expression (12).

## DISCUSSION

La-related protein 1 (LARP1) belongs to the ubiquitous family of La-related proteins, which controls the metabolism of many RNAs in the cell (46). LARP1 has been implicated in regulating mRNA stability and localization (2–4,17). Levels of LARP1 are upregulated in breast, cervical, and liver cancers and the protein is tumorigenic (12). Until now the detailed mechanism of LARP1 function has remained largely uncharacterized.

LARP1 has been linked to ribosome biogenesis via interaction with TOP mRNAs, which encode ribosomal proteins and translation factors, and whose translation is regulated by mTOR kinase (5,16). It has been postulated that LARP1 acts downstream of mTORC1 and as a direct interactor with TOP mRNAs. However, the exact site of its interaction with these transcripts has remained elusive (5). Here, we report the first direct RNA-binding ability of the LARP1-specific RNA binding domain contained within the DM15 boxes and present structural data of this domain that provides insight into its RNA binding functions and begins to address the mechanism of its regulation by mTOR.

Each La-related protein has a cognate RNA binding sequence. Until now, the cognate binding sequence of LARP1 has remained unclear. We have shown that the LARP1-specific DM15 region, a sequence that is highly conserved among LARP1 proteins, but does not appear anywhere else in the proteome, specifically binds the oligopyrimidine-rich sequence present in the 5′TOP of RPS6, but not the 5′TOPs of PABPC1 or RPL13A. Our observations are consistent with studies that have found varied LARP1 dependence on TOP mRNA translation (5,12,16). It is likely that, in the context of the entire LARP1 protein under different physiological states, different RNA binding surfaces might be revealed upon conformational change induced by post-translational modification or by other binding partners to modulate this RNA binding behavior. A deeper understanding of LARP1 binding selectivity awaits elucidation of the LARP1 phospho-interactome in benign and malignant cells.

The structure of the DM15 region from human LARP1 suggests that its unique amino acid sequence has evolved into three HEAT-like helix-turn-helix folds for RNA binding. Thus, while the global fold is not unique, the surface amino acids and lengths of the repeats are novel. As LARP1 has been implicated in cervical, breast, and liver cancers through its interaction with cancer-sustaining mRNAs, the DM15 repeat is a potential cancer drug target. Further, repeat proteins typically utilize their concave surfaces for interactions with substrates or binding partners, suggesting LARP1 has repurposed a commonly used protein-protein-mediating fold for RNA binding.

With the exception of Ascomycota fungi, the DM15 region in all eukaryotic LARP1 sequences is composed of three distinct but related repeats of A, B and C types (Figure 3, Supplementary Figure S4). A likely evolutionary scenario to explain this situation is the expansion of a single motif in the ancestral protein followed by the concerted evolution of each repeat type to achieve specialization. In the Ascomycota lineage (but not for other fungi lineages), selection pressure on the primary sequence of the C repeat (and also to some extent of the A and B repeats) was released and these sequences were allowed to drift from ancestral sequences. Despite this loss of primary sequence, the overall Ascomycota DM15 structure is predicted to be conserved (data not shown). These observations strongly suggest that Ascomycota LARP1 possess a neofunctionalized DM15, that might be functionally distinct from other eukaryotic LARP1 proteins; the absence of conservation of key amino acids shown here to be critical for RNA-binding (R840 and Y883, Figures 1, 3 and Supplementary Figure S4) may indicate that Ascomycota DM15 is no longer an RNA-binding motif.

**Figure 6.**
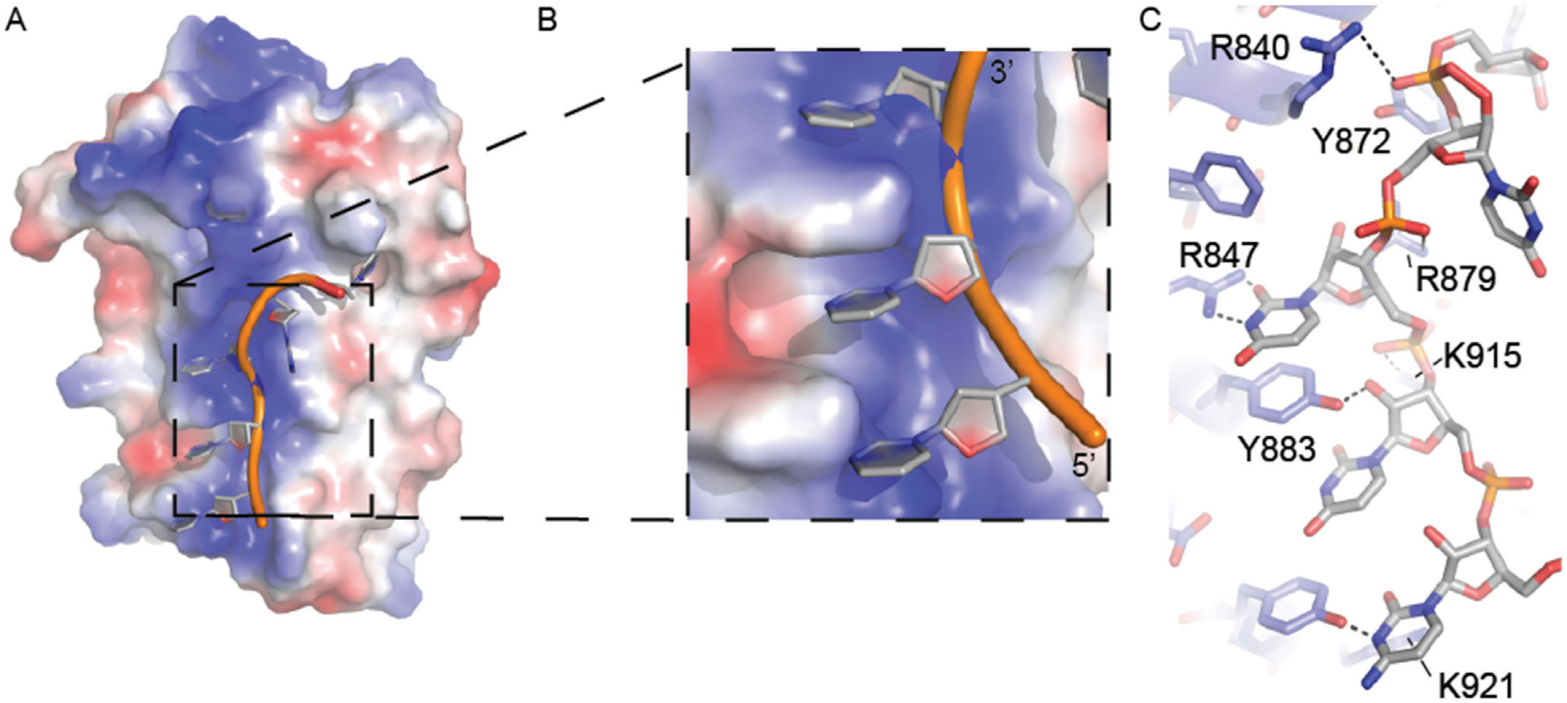
Modelling of single-stranded oligopyrimidine RNA with conserved amino acids on the concave surface of the DM15 region. (**A**) A poly-pyrimidine RNA sequence (CUUUU) was modelled onto the positive patch on the structure of DM15 based on shape and surface complementarity, in addition to hydrogen bonding potential and sulfate ion positioning. (**B**) A zoomed view of the docked RNA boxed in panel (A). (**C**) The specific amino acids used to facilitate RNA docking in the structure are labelled. An additional residue, Y911 (shown in Figure 5) was also used to position the 5′ phosphate of the third residue. Predicted hydrogen bonds shown as black dashed lines.

The structure of the DM15 region reveals a positively charged patch containing conserved residues. Combined with the biochemical binding and mutagenesis data, it is tempting to speculate that this surface of the protein interacts directly with RNA. While it is uncertain that LARP1 exists in the cell as a dimer, the crystallization of the DM15 region as a pseudo-symmetric dimer revealed a positively charged cleft comprised of the concave surfaces of two monomers, which allowed us to hone in on the probable RNA-binding amino acids. We reasoned that the co-crystallized sulfate ion mimics the interactions of the phosphate backbone of a single-stranded RNA binding with an electrostatically positive surface. Therefore, the position of the co-crystallized sulfate ion, in addition to the pattern of highly conserved hydrogen bonding donors and acceptors lining the patch, were used to position a short polypyrimidine sequence on the concave surface of the monomer (Figure 6). Since the DM15 construct interacts with neither the poly(U) oligonucleotide nor the RPS6 ΔTOP, we predict that the DM15 region of LARP1 interacts with both the polypyrimidine and GC-rich regions of the 5′ end of the RPS6 untranslated region, which are characteristic of 5′TOPs (18); nuclease protection assays (Supplementary Figure S1) are consistent with this hypothesis.

In addition to binding RNA, LARP1 interacts with mTOR (16), the serine/threonine kinase that integrates signals emanating from external cues to control cell growth and proliferation via consensus TOR-signalling (TOS) motifs (47,48). mTOR exists in two complexes, mTORC1 and mTORC2, which are comprised of different subunits that impart substrate specificity for downstream signalling and play roles in cellular localization and complex assembly (49). A putative TOS-like sequence appears within the vertebrate LARP1a primary structure in amino acids 928–931, which are contained within the loop connecting α7 and α8 in the crystallized DM15 construct (Supplementary Figure S6). The sequence of the putative TOS motif in LARP1 is consistent with other identified TOS-like motifs (47). Some of the amino acids in the putative LARP1 TOS-like sequence are conservative substitutions of canonical TOS motifs. For example, positions 1 and 3 in the LARP1 TOS-like motif contain leucine and isoleucine, respectively, while the consensus amino acids in these positions in most TOS-motifs are phenylalanine and methionine (47). Mutagenesis demonstrated that the substitution of leucine for phenylalanine at position 1 and isoleucine for methionine at position 3 of the TOS sequence still supports mTORC1 activity (47).

The mTORC1 complex is an obligate dimer (50) and contains Raptor, a large protein that recognizes the TOS motifs of mTOR substrates (47,48,51). The 3D structure of mTORC1 elucidated by electron microscopy reveals a symmetric dimer of the subunits of the complex organized around a cleft of ∼40 × 28 Å (50). Interestingly, the putative TOS sequences within the non-crystallographic dimer of the human DM15 crystal structure presented here are approximately 50 Å apart. Therefore, if LARP1 exists as a dimer in the cell with the DM15 region at its dimerization interface, each TOS motif in the conserved DM15 region can be recognized by Raptor simultaneously (Supplementary Figure S7) to position the potential phosphorylatable amino acids in LARP1 (52) within the active sites of mTORC1 for modification. Many monomeric substrates of mTOR exist in the cell (52), however, indicating that LARP1 can be a substrate of mTORC1 regardless of its oligomeric state.

Through its RNA- and mTORC1-binding abilities, LARP1 plays an important role in the regulation of translation and ribosome biogenesis (5,16). The structure and modelling of the LARP1-specific C-terminal motif, the DM15 region, reveals structural insights into these cellular functions, and other RNA binding activities of LARP1, while also providing the first view of a La-related protein family-specific domain. By visualizing the organization of the key amino acids predicted to mediate mRNA binding by LARP1, we provide potential therapeutic avenues for controlling the pathogenic RNA binding activity of LARP1 in cancer.

## ACCESSION NUMBERS

Structure factors and coordinates have been deposited in the protein data bank under accession 4ZC4 and 5C0V.

## Supplementary Material

SUPPLEMENTARY DATA
