# Supplementary material for: The La-related protein 1-specific domain repurposes HEAT-like repeats to directly bind a 5′TOP sequence
Source: Nucleic Acids Res. 2015 Jul 22;43(16):8077–88. doi: 10.1093/nar/gkv748 (PMC4652764; doi:10.1093/nar/gkv748)
Supplement: SUPPLEMENTARY DATA [file supp_43_16_8077__index.html]

The La-related protein 1-specific domain repurposes HEAT-like repeats to directly bind a 5′TOP sequence — SUPPLEMENTARY DATA 

# The La-related protein 1-specific domain repurposes HEAT-like repeats to directly bind a 5′TOP sequence

## SUPPLEMENTARY DATA

- SUPPLEMENTARY DATA
