# Supplementary material for: The La-related protein 1-specific domain repurposes HEAT-like repeats to directly bind a 5′TOP sequence
Source: Nucleic Acids Res. 2015 Jul 22;43(16):8077–88. doi: 10.1093/nar/gkv748 (PMC4652764; doi:10.1093/nar/gkv748)
Supplement: SUPPLEMENTARY DATA [file supp_gkv748_supplementary-material-rev3sm.pdf]

The LARP1-specific domain DM15 repurposes HEAT-like repeats to directly bind a 5'TOP sequence

Roni M. Lahr, Seshat M. Mack, Annie Héroux, Sarah P. Blagden, Cécile Bousquet-Antonelli, Jean-Marc Deragon, Andrea J. Berman

Supplementary figure legends

Supplementary figure 1. The DM15 motif binds two regions of the RPS6 5' TOP sequence. (**a**) The oligonucleotide sequence used in this experiment (RPS6-20mer) compared to the sequence of the entire 5'TOP from RPS6 (RPS6-42mer) for reference. Regions of DM15 protection are underlined according to experiment: red, RNase A; blue, RNase T1. (**b**) RNase T1 probing of a complex of the DM15 construct and 5'-end radiolabeled RPS6-20mer. Blue bracket indicates region of protection. T1 and OH ladders are included for orientation. (**c**) RNase A probing of a complex of the DM15 motif and RPS6-20mer. Titration of RNase A of RPS6-20mer alone (-) and pre-bound to the DM15 construct (+). Red bracket indicates region of protection.

Supplementary figure 2. Two molecules of DM15 can bind RPS6 sequentially. (**a**) UV crosslinking of DM15 to γ-^{32}P-labeled A_{16} and RPS6-20mer RNA oligonucleotides. (**b**) Gel filtration analysis of protein standards, DM15 construct alone, RPS6-20mer oligonucleotide (3), and DM15 construct + RPS6-20mer oligonucleotide. Absorbance at 280 nm, black lines; absorbance at 254 nm, grey lines. mAU, milliAbsorbance units as reported by the Akta Pure (GE Healthcare Lifesciences). X-axis, elution volume (void volume ~ 40 mL).

Supplementary figure 3. Superposition of the non-crystallographic symmetry mates of DM15. (**a**) Superposition of the four non-crystallographic symmetry mates of the DM15 construct in the asymmetric unit of each structure determined (native and SeMet). The copies superimpose with an RMSD of 0.284-1.106 Å over 142-144 Cαs. Each copy is shown as a different color: SeMet (yellow, green, cyan, and magenta); native (salmon, grey, purple, orange). The N- and C-termini of the constructs are labeled. (**b**) Superposition of the two dimers in the asymmetric unit for both the native and SeMet data. The copies superimpose with an RMSD of 0.302-1.115 Å over the four dimers observed in the two structures determined.

Supplementary figure 4. Figure 3. Alignment of the LARP1 DM15 region from plant, protista, fungi and animal species. Colored boxes indicate the position of the A, B, and C repeats, respectively, while black and grey boxes indicate the position of the eight alpha helices as determined from the crystal structure of the human protein. The intensity of the sequence coloring is proportional to the level of conservation. The species code is the following: **Animal:** Aim: *Ailuropoda melanoleuca*, Am*: Apis mellifera*, Bf: *Branchiostoma floridae*, Bt: *Bos Taurus*, Cb: *Caenorhabditis briggsae*, Ce: *Caenorharbditis elegans*, Cf: *Canis familiaris*, Ci: *Ciona intestinalis*, Cs: *Capitella sp.*, Dm: *Drosophila melanogaster***,** Dp: *Daphnia pullex*, Dr: *Danio rerio*, Ec: *Equus caballus*, Fr: *Fugu rubripes*, Gg: *Gallus gallus*, Hs: *Homo sapiens*, Lg: *Lottia gigantae*, Md: *Monodelphis domestica*, Mm: *Mus musculus*, Nav: *Nasonia vitripennis*, Rn: *Rattus norvegicus*, Ta: *Trichoplax adhaerens*, Tg: *Taeniopygia guttata*, Xt: *Xenopus tropicalis*. **Fungi:** *Aspwe: Aspergillus wentii*, Aurpu: ***Aureobasidium pullulans*****,** *Bacci: Backusella circina, Batde: Batrachochytrium dendrobatidis, Botci: Botrytis cinerea, Chove: Choiromyces venosus, Clagr: Cladonia grayi, Coere: Coemansia reversa, Conco: Conidiobolus coronatus, Gloin: Rhizophagus Irregularis, Gymau: Gymnascella aurantiaca, Lichy: Lichtheimia hyalospora, Mucci: Mucor circinelloides, Neopa: Neofusicoccum parvum*, Neucr: *Neurospora crassa, Pire: Piromyces sp., Phybl: Phycomyces blakesleeanus, Rhimi: Rhizopus microsporus, Sclsc: Sclerotinia sclerotiorum, Spoth: Sporotrichum thermophile, Theau: Thermoascus aurantiacus, Trepe: Trematosphaeria*

pertusa, Umbra: Umbelopsis ramanniana, Wilmi: Wilcoxina mikolae, Xylhe: Xylona heveae. ***Plant and algae:*** At: *Arabidopsis thaliana*, Cr: *Chlamydomonas reinhardii*, Ms: *Micromonas sp.*, Ol: *Ostreococcus lucimarinus*, Os: *Oryza sativa*, Php: *Physcomitrella patens*, Pt: *Populus trichocarpa*, Sb: *Sorghum bicolor*, Sm: *Selaginella moellendorfii*, Vv: *Vitis vinefera*. **Protista:** Mb: *Monosiga brevicollis*, Dd: *Dictyostelium discoideum*.

Supplementary figure 5. Several pseudo-symmetric interactions maintain the dimerization interface of DM15. (**a**) Intermolecular hydrogen bonds between side chains and backbone atoms. (**b**) Intermolecular ionic interactions. (**c**) Intermolecular hydrophobic interactions. (**d**) Position of R840 at the interface. (**e**) Position of Y883 near the dimerization interface. (**f**) Position of G908, Q909 at the interface. All relevant amino acids are shown as sticks superimposed on a ribbon of the α-carbon trace of each monomer. One monomer is colored cyan, while the other is colored green.

Supplementary figure 6. Putative TOS motif resides in a loop C-terminal to the DM15 repeats. Cartoon diagram of the crystallized DM15 region of LARP1 colored and termini labeled as in Figure 1. The view is approximately 180° rotated about the x-axis, as compared to the view in Figure 1a. The amino acids consistent with the consensus TOS motif are shown as sticks and boxed. Boxed zoom, a rotated view of the putative TOS motif in LARP1. Below, sequence alignment of known potential TOS motifs from selected Raptor-binding proteins. Asterisks indicate amino acids that have been shown to be necessary for functioning as a TOS motif[1].

Supplementary figure 7. The envelopes of the mTORC1 obligate dimer[2] and the DM15 dimer are drawn to scale. The positions of Raptor within mTORC1 are indicated. Green stars represent putative TOS elements in the DM15 motif. Approximate dimensions and distances are shown in Ångstroms. The position of the TOS motifs in the DM15 dimer align with the two copies of Raptor present in the dimer of mTORC1, suggesting that Raptor can potentially recognize the TOS motifs of two DM15 regions simultaneously.

References for Supplemental figure legends

1. Lee, V.H., Healy, T., Fonseca, B.D., Hayashi, A. & Proud, C.G. Analysis of the regulatory motifs in eukaryotic initiation factor 4E-binding protein 1. *FEBS J* **275**, 2185-99 (2008).
2. Yip, C.K., Murata, K., Walz, T., Sabatini, D.M. & Kang, S.A. Structure of the human mTOR complex I and its implications for rapamycin inhibition. *Mol Cell* **38**, 768-74 (2010).

A RPS6-20mer: 5’ CCUCUUUUCCGUGGCGCCUC 3’
RPS6-42mer: 5’ CCUCUUUUCCGUGGCGCCUCGGAGGCGUUCAGCUGCUUCAAG

B

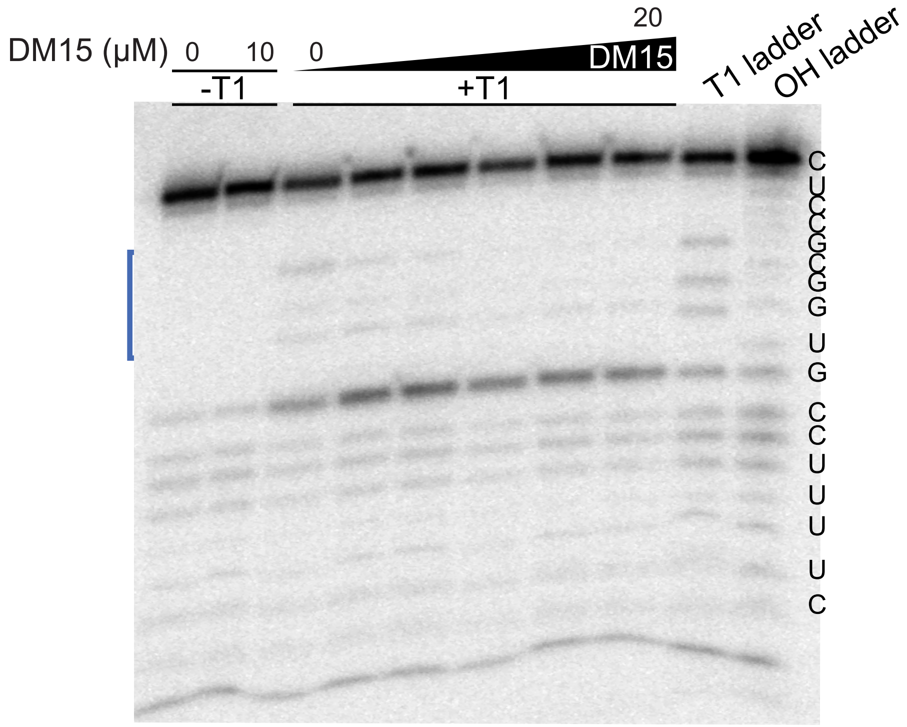

C

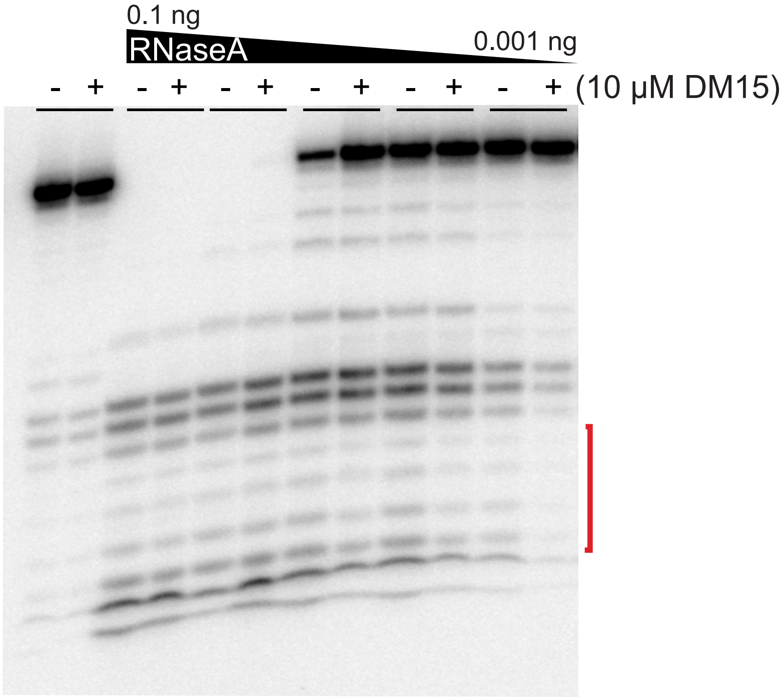

S. 1

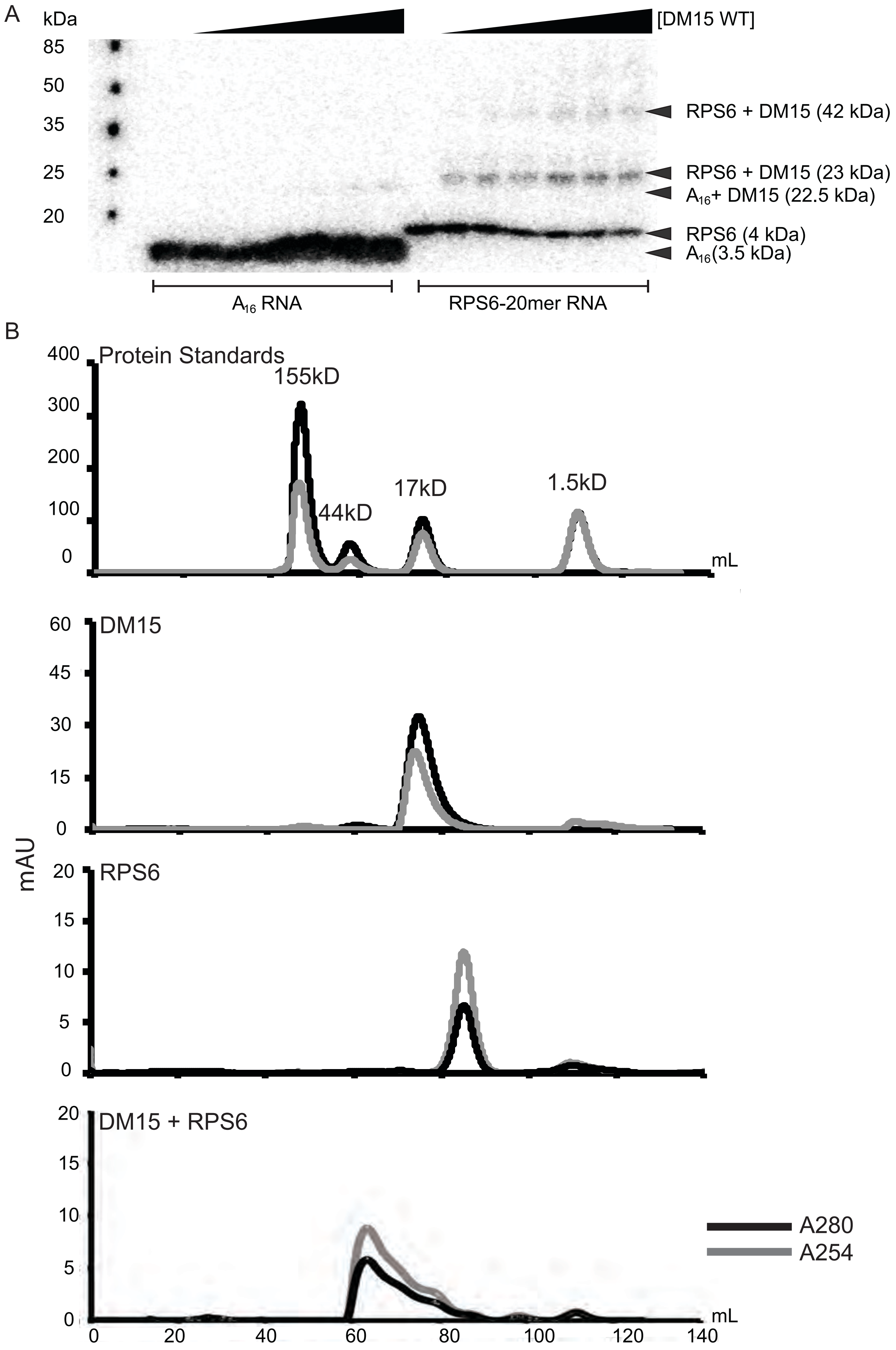
A
kDa
85
50
35
25
20
[DM15 WT]
RPS6 + DM15 (42 kDa)
RPS6 + DM15 (23 kDa)
A_{16}+ DM15 (22.5 kDa)
RPS6 (4 kDa)
A_{16}(3.5 kDa)
A_{16} RNA
RPS6-20mer RNA
B
Protein Standards
155kD
44kD
17kD
1.5kD
DM15
RPS6
DM15 + RPS6
mAU
mL
A280
A254
0
20
40
60
80
100
120
140

S. 2

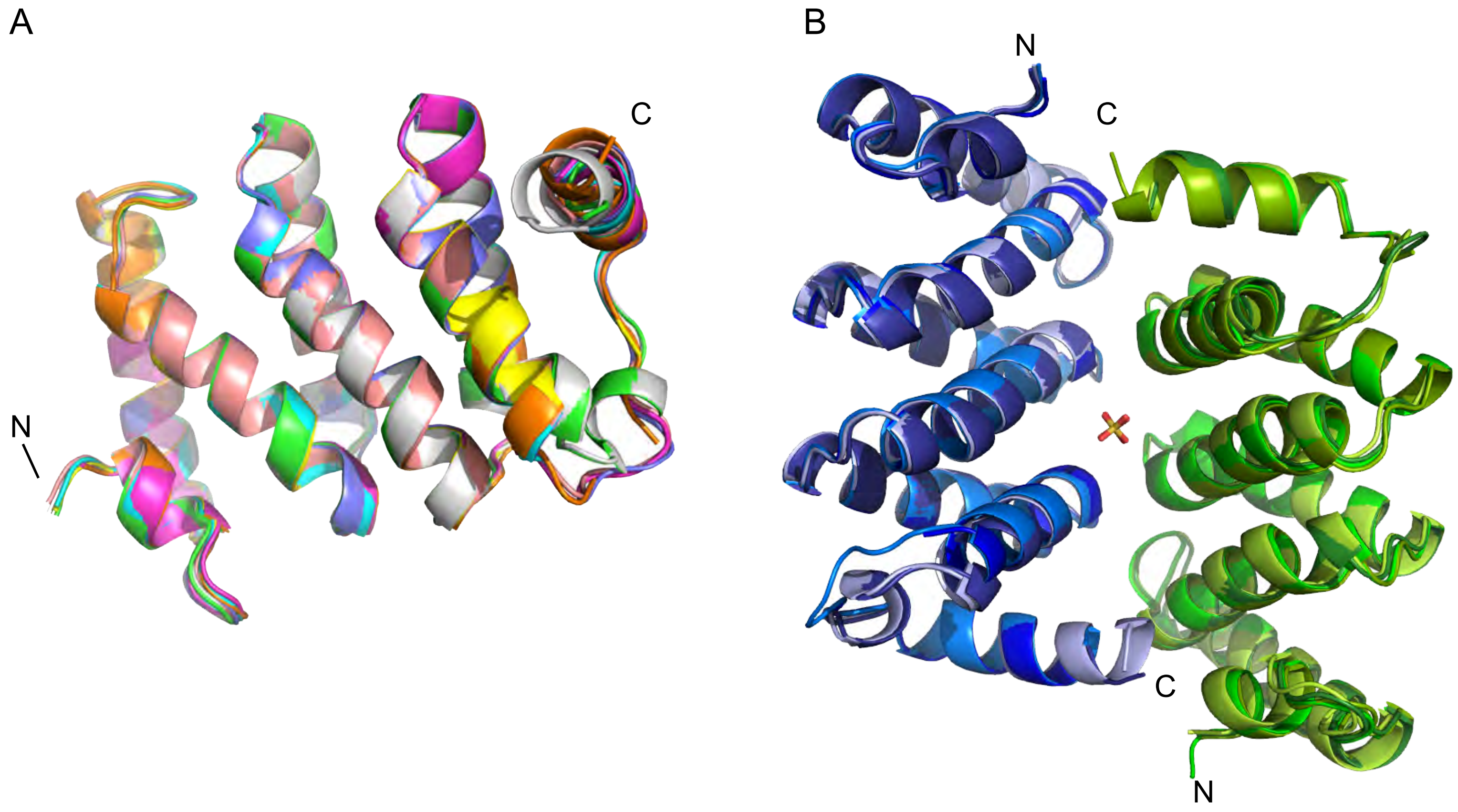

S. 3

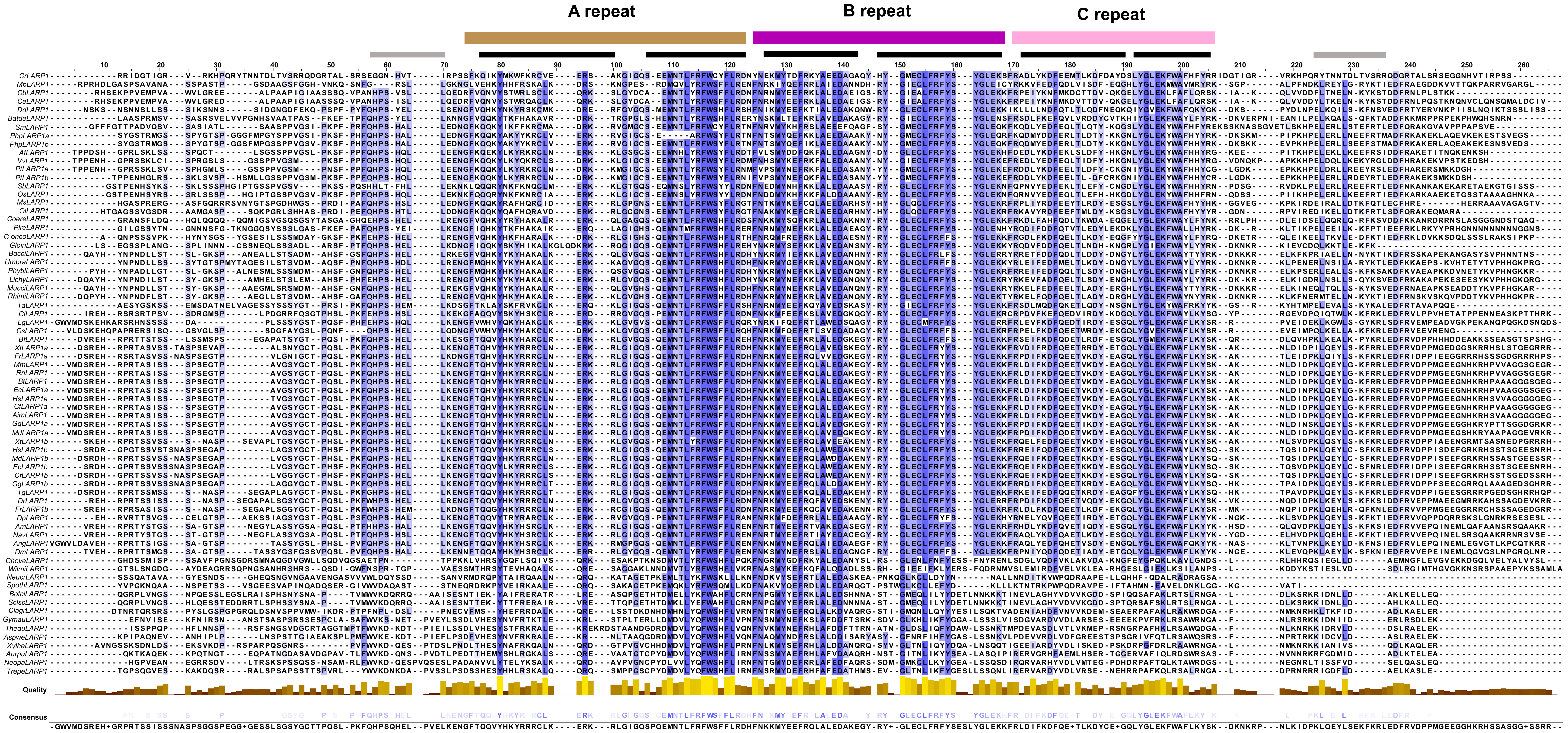

S. 4

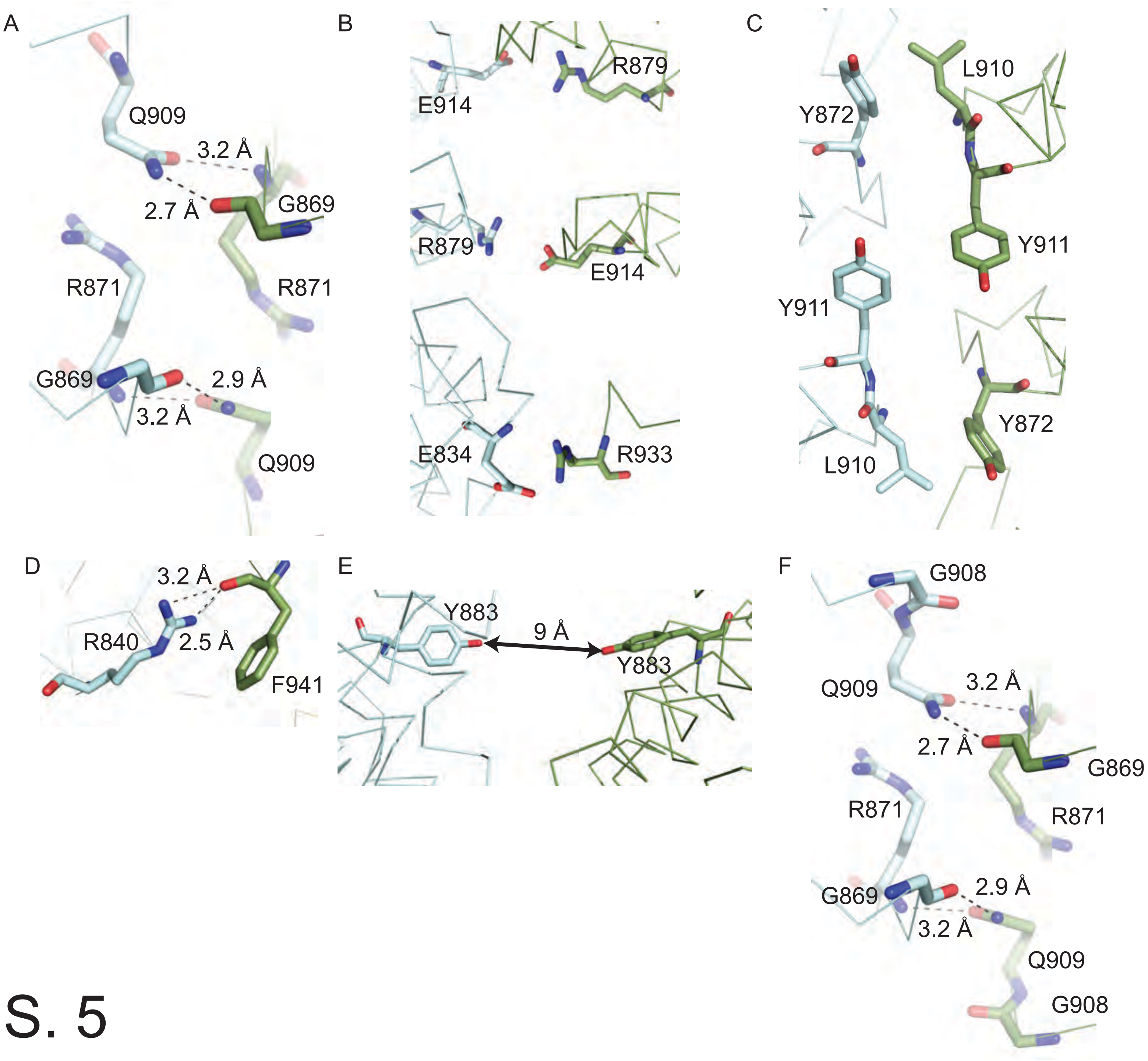
A
Q909
3.2 Å
2.7 Å
G869
R871
R871
G869
2.9 Å
3.2 Å
Q909
B
E914
R879
R879
E914
E834
R933
C
Y872
L910
Y911
Y911
Y872
L910
D
3.2 Å
R840
2.5 Å
F941
E
Y883
9 Å
Y883
F
G908
Q909
3.2 Å
2.7 Å
G869
R871
R871
G869
2.9 Å
3.2 Å
Q909
G908

S. 5

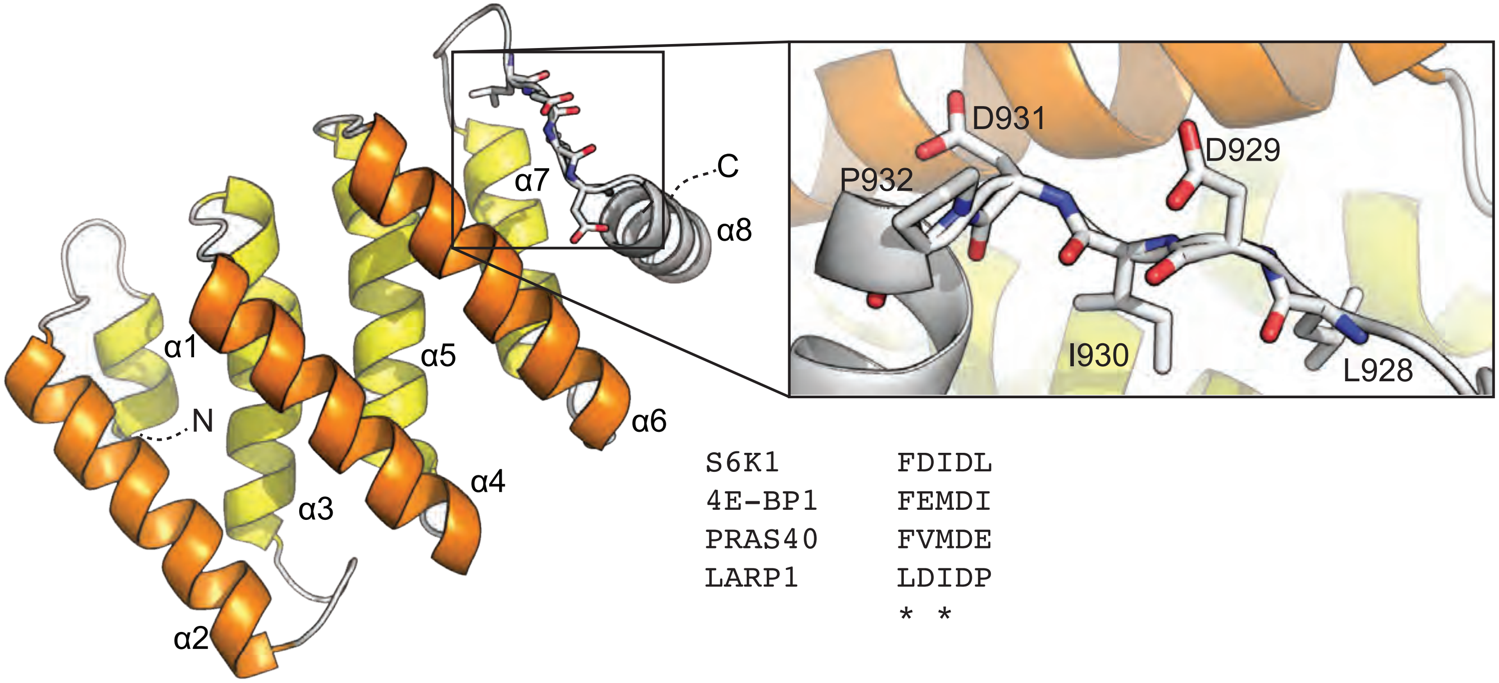

D931
D929
P932
I930
L928
α1
α2
α3
α4
α5
α6
α7
α8
N
C

```
S6K1      FDIDL
4E-BP1    FEMDI
PRAS40    FVMDE
LARP1     LDIDP
          * *
```


S. 6

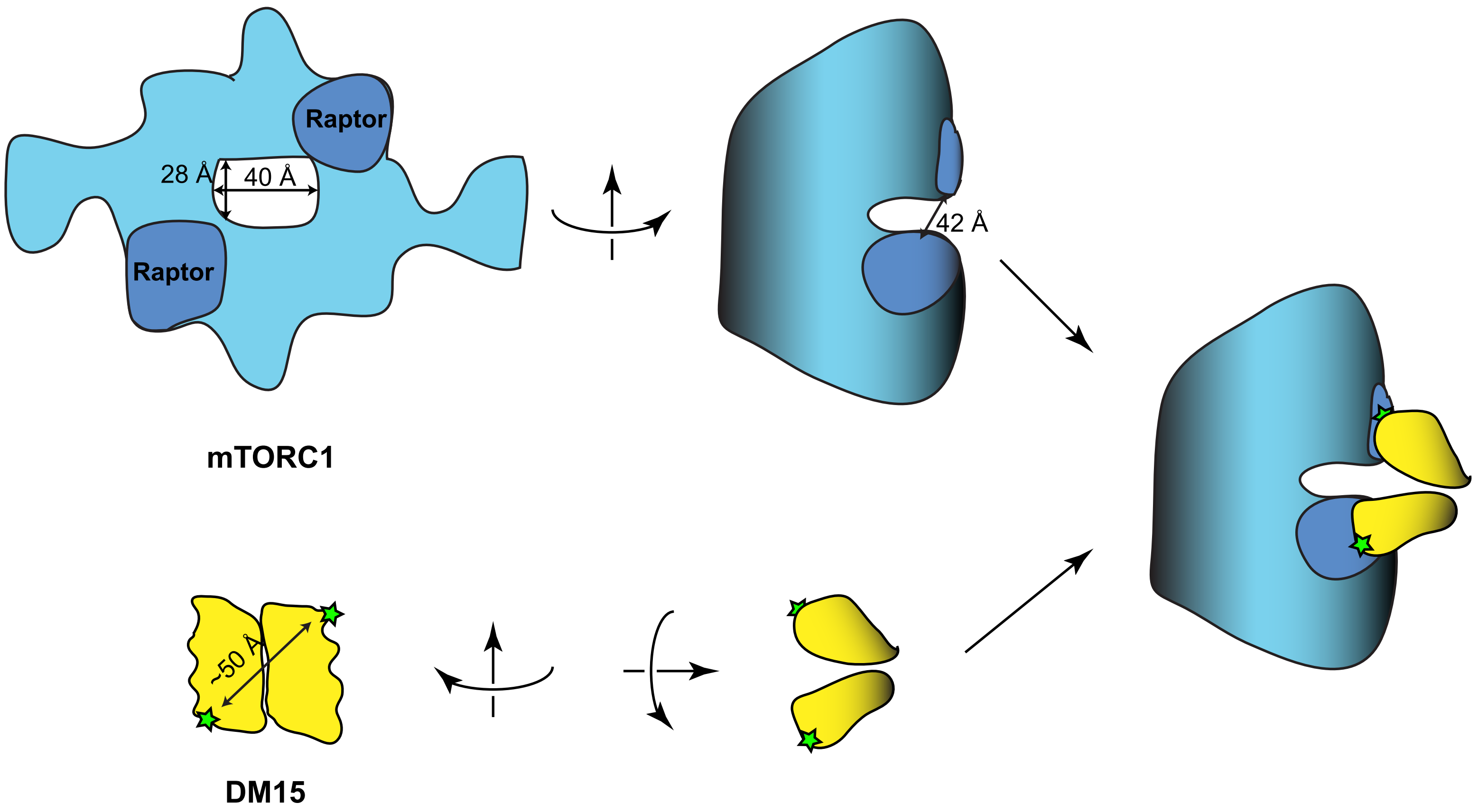

S.7
